# Pre- and Postnatal Polychlorinated Biphenyl Concentrations and Longitudinal Measures of Thymus Volume in Infants

**DOI:** 10.1289/ehp.1104229

**Published:** 2012-01-03

**Authors:** Todd A. Jusko, Dean Sonneborn, Lubica Palkovicova, Anton Kocan, Beata Drobna, Tomas Trnovec, Irva Hertz-Picciotto

**Affiliations:** 1 Epidemiology Branch, National Institute of Environmental Health Sciences, National Institutes of Health, Department of Health and Human Services, Research Triangle Park, North Carolina, USA; 2 Division of Epidemiology, Department of Public Health Sciences, School of Medicine, University of California–Davis, Davis, California, USA; 3 Department of Environmental Medicine, and; 4 Department of Toxic Organic Pollutants, Slovak Medical University, Bratislava, Slovakia

**Keywords:** atrophy, cohort, epidemiology, immune, Roma, T cell

## Abstract

Background: Previously, we reported an association between higher maternal polychlorinated biphenyl (PCB) concentrations and smaller thymus volume in newborns in a birth cohort residing in eastern Slovakia.

Objective: In the present report we address whether thymus volume at later ages is influenced by prenatal and early postnatal PCB exposure.

Methods: At the time of delivery, 1,134 mother–infant pairs were enrolled. Maternal and 6- and 16-month infant blood samples were collected and analyzed for 15 PCB congeners. Thymus volume was measured in infants shortly after birth and at ages 6 and 16 months using ultrasonography.

Results: Higher maternal PCB concentration was associated with reduced thymus volume at birth [a 0.21 SD reduction in thymus volume for an increase in total maternal PCB concentration from the 10th to the 90th percentile; 95% confidence interval (CI): –0.37, –0.05], whereas maternal PCB concentration was not predictive of 6- and 16-month thymus volume. Six-month infant PCB concentration was associated with a 0.40 SD decrease in 6-month thymus volume (95% CI: –0.76, –0.04). There was also some suggestion that thymus volume at 16 months was positively associated with concurrent infant PCB concentration.

Conclusions: The potential adverse effects of *in utero* PCB exposure on thymic development may extend beyond the neonatal period. Results from this highly exposed cohort provide suggestive evidence that postnatal PCB concentrations may be influential, but a smaller set of 6-month PCB measurements limited statistical power at that time point. Implications regarding impaired immunologic maturation or long-term clinical implications remain to be determined.

Polychlorinated biphenyls (PCBs), a class of 209 congeners with two linked phenyl rings and variable chlorination in different sites, were introduced in 1929. Before being banned in most industrialized countries in the late 1970s because of their persistence, PCBs were widely used as dielectric fluids in electrical transformers and capacitors and as heat exchangers or hydraulic fluids (Agency for Toxic Substances and Disease Registry 2000). Although concentrations of PCBs, dioxin, and dibenzofurans in humans are declining (Fangstrom et al. 2005; Norén 1988; Schecter et al. 2005), their improper disposal has resulted in low-level environmental contamination worldwide. Particularly affected are populations with significant contamination from past manufacturing and improper disposal (Hansen et al. 2003; Kocan et al. 2001; Park et al. 2007) or those populations whose diet includes consumption of PCB-contaminated fish or seafood (Dallaire et al. 2004; Fangstrom et al. 2005).

PCBs are structurally related to 2,3,7,8-tetrachlorodibenzo-*p*-dioxin (TCDD, or “dioxin”), and depending on their particular chlorine substitution pattern, some congeners may produce health effects similar to TCDD (i.e., the so-called “dioxin-like” PCB congeners) (Van den Berg et al. 2006). In terms of their potential developmental immunotoxicity, TCDD and dioxin-like PCBs have well-established effects on the immune system, one of which is thymic atrophy, an outcome observed in all species evaluated after TCDD exposure (Lawrence and Kerkvliet 2006). The thymus is a lymphoid organ responsible for T-cell differentiation and maturation and is seeded by lymphocyte stem cells that originate from the liver and yolk sac during gestation and from bone marrow postnatally (Moore and Owen 1967). Although the exact mechanism by which TCDD-induced thymic atrophy occurs is not known, potential mechanisms include a reduction in prothymocyte stem cells in the bone marrow and an inhibition of thymocyte maturation (Silverstone et al. 1994), and induction of apoptosis in thymocytes (McConkey et al. 1988).

Although the immunologic effects of dioxins and PCBs are well documented in experimental studies, potential effects on the human immune system are less well studied. A previous analysis from this cohort reported smaller thymus volumes at birth in relation to higher maternal serum PCB concentrations (Park et al. 2008), the first such study in humans. However, it is not known whether *in utero* and/or postnatal PCB exposures are associated with thymus volume later in infancy. The present study expands our investigation longitudinally to an examination of infant thymus volume at 6 and 16 months of age, in a population with substantial PCB exposure.

## Materials and Methods

*Study population and follow-up.* Pregnant women were recruited for this study at the time of their admittance to the hospital for delivery (Hertz-Picciotto et al. 2003). Recruitment occurred between 2002 and 2004 in two districts of eastern Slovakia: Michalovce, a region with substantial environmental contamination due to past manufacturing (Kocan et al. 2001); and Svidnik, a region located approximately 70 km to the northwest, with less environmental contamination and lower serum PCB concentrations among inhabitants (Petrik et al. 2006). Because each district has only one hospital, the vast majority of births to residents in these two districts occur in these hospitals. We excluded mothers who had more than four previous births, who were < 18 years of age at the time of delivery, who reported residing < 5 years in either district, or who had a major illness during pregnancy. Infants with severe birth defects were also excluded. Trained nursing staff at each hospital explained the study procedures, and all participants gave written informed consent.

All forms of communication were in the Slovak language. In total, 1,134 women were enrolled (811 in Michalovce and 323 in Svidnik). During the hospital stay, each infant underwent an ultrasound examination of the thymus. The study protocol was approved by institutional review boards at the University of California–Davis and the Slovak Medical University in Bratislava.

When the infants were approximately 6 and 16 months of age, mother–infant pairs were invited back to the Department of Pediatrics at their local hospital in either Michalovce or Svidnik, where blood was collected for PCB analysis and infants again underwent ultrasound examinations (described below). At the infants’ age 6 months, 971 mother–infant pairs were still participating in the study (86%), and at age 16 months, 887 (78%) were still participating.

*Specimen collection.* At the time of delivery, two 9-mL Vacutainer tubes were used to collect maternal blood, and at the 6- and 16-month follow-ups, approximately 9 mL of blood was collected from the infant. Details on the processing, transport, and storage of specimens and the isolation of serum have been published elsewhere (Jusko et al. 2010).

*PCB and lipid measurement.* The concentrations of 15 PCB congeners [International Union of Pure and Applied Chemistry (IUPAC) PCB congeners 28, 52, 101, 105, 114, 118, 123+149, 138+163, 153, 156+171, 157, 167, 170, 180, and 189] were determined in maternal and 6- and 16-month serum samples. The procedure for determination of PCB concentrations involved extraction, cleanup, and quantitation by high-resolution gas chromatography with electron capture detection; specifics of the analysis are detailed elsewhere (Conka et al. 2005; Jusko et al. 2010). PCB congeners 123+149, 138+163, and 156+171 coeluted on the gas chromatograph column. The Department of Toxic Organic Pollutants at the Slovak Medical University in Bratislava performed the laboratory analyses. This laboratory has participated in intercalibration studies organized by the World Health Organization (WHO) and the German Agency for Occupational and Environmental Medicine (Deutsche Gesellschaft fuer Arbeitsmedizine und Umweltmedizin e.V.). Total serum lipids were measured at a biochemical laboratory accredited by the Slovak National Accreditation Service located at the Ministry of Defense military hospital in Bratislava, and total lipid concentrations were estimated using the enzymatic summation method (Akins et al. 1989).

Maternal PCBs were analyzed for 1,104 women. At age 6 months, 249 infant specimens were selected for analysis based on their corresponding maternal PCB concentration. Specifically, 6-month infant specimens were randomly sampled within strata defined by total maternal PCB concentrations: *a*) < 75th percentile, *b*) between 75th and 85th percentiles, *c*) between 85th and 95th percentiles, and *d*) > 95th percentile (Jusko et al. 2010). At 16 months of age, 831 infant serum specimens were analyzed (all available specimens).

*Thymus measurement.* Thymus volume was measured by four radiologists from Michalovce and two radiologists from Svidnik using a sonographic scanner [Esaote 580 FD Caris plus (convex probe 7.5 MHz) in Michalovce; Esaote AU 5 Harmonic (convex probe 7.5 MHz) in Svidnik; both from Esaote, Genoa, Italy]. The radiologists were unaware of maternal and infant PCB concentrations. Measurements of the thymus derived from sonographs are regarded as reliable, especially in young children (Hasselbalch et al. 1996, 1997). A transsternal approach was used to measure the maximal transverse diameter (width) of the thymus, and in the plane perpendicular to this width, the largest sagittal area (longitudinal scan plan) was also measured. These two measurements were multiplied to obtain a “thymic index,” a proxy of thymus volume. The thymic index has been used as a surrogate for thymus volume, because it is strongly correlated (*r* ≥ 0.80) with the actual volume and weight of the thymus (Hasselbalch et al. 1996) (hereafter, the thymic index is referred to as thymus volume). Thymus volume was measured in 1,074 newborns, 940 children 6 months of age, and 820 children 16 months of age, for whom 1,020 (95%), 241 (26%), and 806 (98%) had PCB measurements.

*Other data collection.* Maternal questionnaire. During the 5-day hospital stay after delivery, trained nursing staff administered a questionnaire that obtained information on lifestyle and living environment, past pregnancies and medical conditions, medication use during and before pregnancy, and sociodemographic data, including ethnicity. Ethnicity was categorized into two groups: Slovakian/other eastern European or Romani. Romani ethnicity was attributed to the mother if the ethnic origin of either of her parents was Romani, the Romani language was spoken at the home of the child, or the mother was planning to raise her child with the Romani language. Romani ethnicity was subsequently independently confirmed by a Slovak member of the research team who used additional information such as the family’s last name to confirm ethnicity. Data on maternal smoking and alcohol use during and in the 3 months before pregnancy were also collected. History of maternal illness was also obtained, which included respiratory symptoms, asthma, or allergy during pregnancy or in the 3 months before pregnancy. At the 6- and 16-month follow-up visits, mothers again completed a questionnaire that updated demographic and lifestyle information and data on current maternal smoking and collected data on the child’s day care attendance and breast-feeding.

Infant medical records. The infant’s medical records were abstracted at birth and 6 and 16 months to obtain birth weight, gestational age (weeks), and the child’s weight taken at well- and sick-child visits with their pediatricians. Because these office visits did not necessarily coincide with the dates of their thymus measurements at 6 and 16 months, we interpolated linearly the two weights closest in time both before and after the thymus measurement date to estimate the child’s weight at the time of thymus measurement.

*Statistical methods.* Selection of PCBs for statistical models. We selected maternal and infant PCB congeners for inclusion in our statistical models if at least 80% of the measurements were above the limit of detection (LOD) in maternal and 6- and 16-month serum samples. These were congeners 138+163, 153, 170, and 180. When an individual congener value was below the LOD, we assigned the value as the LOD divided by the square root of 2 (Lubin et al. 2004). The sum PCB variable is therefore the arithmetic sum of these four congeners.

Multivariate methods. To eliminate differences in thymic volume measures attributable to different examiners, we standardized thymic volume by examiner and within each time point (birth and 6 and 16 months of age). We fit repeated measures models that included a repeated measure of standardized thymus volume at birth and 6 and 16 months of age as the dependent variable. One set of models focused on maternal PCB concentration in relation to all three thymus volume measurements; the other examined time-varying PCB exposure based on maternal and 6- and 16-month infant PCB concentrations as the exposure of interest. Continuous PCB concentrations (per lipid basis, in nanograms per gram of lipid) were modeled as natural log values to reduce the influence of extreme values, and preliminary, age-specific models showed that the PCB–thymus volume association was adequately modeled with a linear term. To allow for age to modify the associations between PCB concentrations and thymus volume, we included a PCB × age interaction term, where age was a categorical variable (0, 6, and 16 months, corresponding to the month of assessment). We used directed acyclic graphs to select covariates for our model (Greenland et al. 1999), which included ethnicity (Romani/other), infant sex, district of residence (Svidnik/Michalovce), infant weight (kilograms), and duration of exclusive breast-feeding (months). Infant weight was modeled as a time-varying covariate using the recorded birth weight and the interpolated weight at 6 and 16 months of age, as described above. Duration of exclusive breast-feeding was also parameterized as a time-varying covariate and included an interaction with categorical time, so that separate effects could be estimated at 6 and 16 months of age. Duration of exclusive breast-feeding values at birth were set equal to 0 (because women were not yet breast-feeding), and values at 6 and 16 months corresponded to the duration of exclusive breast-feeding up until that point in time. All models were fit using the MIXED procedure in SAS (version 9.2; SAS Institute Inc., Cary, NC, USA) with random effects specified for intercept and age at assessment (weeks), with no explicit structure assumed for the covariance among repeated measures (“unstructured covariance”). Because only a portion (*n* = 249) of 6-month infant PCB samples were analyzed, and the specimens selected for analysis were selected based on maternal PCB concentration (stratified random sampling), sampling weights were added for the 6-month time point (weights were set to 1 at birth and 16 months of age). Finally, to facilitate interpretability and for consistency with a previous study of PCBs and infant thymus size from this cohort (Park et al. 2008), we present the results for regression models as the difference in standardized thymus volume [and associated 95% confidence intervals (CIs)] for an increase in PCB concentration from the 10th to the 90th percentile. In addition to our primary model (described above), we limited our study sample to those infants who had thymus measures conducted within the first postnatal week and within 2 weeks of their 6- and 16-month birthdays. Results using the truncated age range are presented alongside our primary model for comparison.

We also conducted two sensitivity analyses to evaluate the role of exclusive breast-feeding duration on PCB concentrations and thymus development. First, we fit 6- and 16-month PCB models without adjustment for breast-feeding to evaluate the role of confounding, and second, we also examined potential heterogeneity by exclusive breast-feeding duration at the 6- and 16-month time points. Results of the sensitivity analyses are reported in text.

## Results

*Baseline sample characteristics.* Table 1 depicts characteristics of the 1,056 mother–infant pairs who contributed at least one observation to the repeated measures model (93% of the original cohort). Nearly three-quarters of these families lived in the Michalovce district, 17% of the mothers were ≥ 30 years of age, 59% had at least 12 years of education, and the vast majority (92%) were married and living with their partners. Just over half (53%) of mothers exclusively breast-fed their infant for at least 3 months.

**Table 1:** Characteristics of infants and mothers who contributed at least one observation to the mixed model (*n* = 1,056).

Characteristic	*n* (%)*a*	
District			
Michalovce		760	(72)
Svidnik		296	(28)
Infant sex			
Male		539	(51)
Female		517	(49)
Gestation length (weeks)			
< 37		25	(2)
37–41		1,001	(95)
≥ 42		30	(3)
Maternal education			
< 12		432	(41)
12–16		562	(53)
≥ 16		62	(6)
Marital status			
Married		972	(92)
Never married		65	(6)
Widowed		4	(0)
Divorced/separated		15	(1)
Months of exclusive breast-feeding			
0 to < 3		495	(47)
3–6		543	(51)
> 6		18	(2)
Ethnicity of child			
Romani		224	(21)
Slovak/eastern European		832	(79)
Maternal age at child’s birth (years)			
18 to < 20		91	(9)
20–30		784	(74)
> 30		181	(17)
**a**Percentages may not sum to 100 because of rounding.			

*PCB concentrations.* Maternal and 6- and 16-month infant sum PCB concentrations (congeners 138+163, 153, 170, and 180) are presented in Table 2, as well as PCB-153 concentrations for purposes of comparison with other populations (Longnecker et al. 2003). The median maternal PCB-153 concentration was 143 ng/g lipid (10th percentile, 62 ng/g lipid; 90th percentile, 369 ng/g lipid) and ranged from 11 to 3,958 ng/g lipid. Compared with maternal serum concentrations, median PCB-153 concentrations were lower in infants at 6 months (98 ng/g lipid) and 16 months (106 ng/g lipid). When infant PCB concentrations were considered separately by duration of exclusive breast-feeding, results showed that infants who were exclusively breast-fed for ≥ 3 months had higher PCB concentrations than those exclusively breast -fed for a shorter duration, both for the sum PCB concentration and for PCB-153.

**Table 2:** PCB concentrations in maternal and 6- and 16-month serum samples of mother–infant pairs contributing at least one observation to the mixed model.*^a^*

Wet weight (ng/mL)															Per lipid (ng/g or ppb)														
PCB measure	*n*	Mean	Min	P10	P25	P50	P75	P90	Max	Mean	Min	P10	P25	P50	P75	P90	Max
Maternal PCB																																		
Sum*b*		966		6.09		0.46		1.76		2.68		4.18		6.92		11.68		153.93		591		49		183		267		415		660		1,110		10,853
PCB-153		966		2.03		0.11		0.61		0.92		1.42		2.29		3.92		56.13		197		11		62		91		143		220		369		3,958
6-month PCB*c*																																		
Exclusively breast-fed < 3 months																																		
Sum*b*		98		1.40		0.02		0.16		0.27		0.53		1.37		3.36		31.29		233		4		26		51		94		232		544		3,027
PCB-153		98		0.51		0.01		0.08		0.11		0.20		0.54		1.13		3.300		85		2		13		20		35		85		189		1,089
Exclusively breast-fed ≥ 3 months																																		
Sum*b*		112		4.68		0.13		0.60		1.74		3.11		5.08		9.30		138.34		821		24		103		278		586		890		1,348		28,540
PCB-153		112		1.64		0.05		0.22		0.57		1.15		1.75		3.30		46.56		288		10		39		91		206		306		503		9,606
All infants																																		
Sum*b*		210		3.19		0.02		0.21		0.42		1.74		3.91		6.51		138.3		553		4		37		87		278		726		1,231		28,540
PCB-153		210		1.12		0.01		0.09		0.17		0.62		1.42		2.10		46.56		195		2		15		33		98		264		396		9,606
16-month PCB																																		
Exclusively breast-fed < 3 months																																		
Sum*b*		311		1.81		0.01		0.19		0.32		0.61		1.33		3.51		74.24		305		2		34		54		104		230		564		11,164
PCB-153		311		0.61		0.00		0.07		0.12		0.24		0.52		1.25		14.00		103		1		12		20		39		85		207		2,036
Exclusively breast-fed ≥ 3 months																																		
Sum*b*		435		4.72		0.01		0.62		1.53		2.89		5.61		10.31		46.84		805		2		114		264		503		942		1,679		10,122
PCB-153		435		1.68		0.00		0.24		0.56		1.04		1.94		3.55		16.21		287		0		43		95		183		341		563		3,504
All infants																																		
Sum*b*		746		3.51		0.01		0.27		0.57		1.66		3.99		7.86		74.24		597		2		49		100		292		707		1,321		11,164
PCB-153		746		1.23		0.00		0.10		0.21		0.62		1.46		2.74		16.21		210		0.5		18		37		106		254		461		3,504
Abbreviations: Max, maximum; Min, minimum; P, percentile. **a**Values reflect the distributions after imputation of values below the LOD. **b**Sum of PCB congeners 138+163, 153, 170, and 180. **c**Weighted to reflect underlying distribution of 6-month PCB concentrations in the cohort.																																		

Lipid-adjusted maternal sum PCB concentration was moderately associated with 6-month infant (Spearman *r* = 0.49) and 16-month infant (Spearman *r* = 0.33) lipid-adjusted sum PCB concentrations. Six- and 16-month infant lipid-adjusted PCB concentrations were strongly associated (Spearman *r* = 0.92). Results were similar when sum PCB concentrations were expressed as wet-weight concentrations.

*Thymus ultrasound measures.* The median (SD) nonstandardized thymus index was 8.7 (4.5) at birth, 14.1 (7.1) at the 6-month follow-up, and 9.4 (7.5) at the 16-month follow-up. Most (85%) neonatal thymus examinations were conducted within the first week of birth, with a maximum delay of 4 weeks. The median age was 26 weeks (range, 24–48 weeks) at the 6-month thymus examination and 70 weeks (range, 66–88 weeks) at the 16-month follow-up.

*PCB concentrations and infant thymus index.* Table 3 shows the results of a repeated measures regression model predicting standardized thymus volume as a function of PCB concentration, adjusting for potential confounding variables. An increase in sum maternal serum PCB concentration from the 10th to the 90th percentile was associated with a 0.21-SD reduction in thymus volume at birth (95% CI: –0.37, –0.05); maternal sum PCB concentrations were not associated with measures of thymus volume at 6 and 16 months of age. Six-month infant PCBs were associated with reduced thymus volume at 6 months of age (a 0.40-SD reduction for an increase in sum serum PCB concentration from the 10th to the 90th percentile), although the CI for this estimate was wide (95% CI: –0.76, –0.04). There was some suggestion that 16-month infant PCB concentration was positively associated with thymus volume assessed at 16 months of age (0.17-SD increase; 95% CI: –0.04, 0.38).

**Table 3:** Adjusted change in infant thymus volume at birth and 6 and 16 months of age in relation to maternal and 6- and 16-month infant PCB concentrations.

Model	*na*	Change in thymus index [SD (95% CI)]*b *
Maternal PCB*c*				
Thymus index at birth				
Primary model*d*		966		–0.21 (–0.37, –0.05)
Truncated age range*e*		951		–0.23 (–0.38, –0.07)
Thymus index at infant 6 months				
Primary model		761		–0.09 (–0.29, 0.11)
Truncated age range		644		–0.14 (–0.26, 0.07)
Thymus index at infant 16 months				
Primary model		721		–0.02 (–0.21, 0.17)
Truncated age range		519		0.09 (–0.13, 0.07)
6-month infant PCB*c*				
Thymus index at 6 months				
Primary model		210		–0.40 (–0.76, –0.04)
Truncated age range		186		–0.54 (–0.94, 0.13)
16-month infant PCB*c*				
Thymus index at 16 months				
Primary model		746		0.17 (–0.04, 0.38)
Truncated age range		538		0.24 (–0.01, 0.49)
**a**Number of observations for each PCB–thymus association; owing to the repeated measure model, the total number of unique observations in each statistical model was always greater. **b**Estimated change in standardized infant thymus volume as PCB concentration increases from the 10th to the 90th percentile. **c**Sum of PCB congeners 138+163, 153, 170, and 180. **d**Adjusted for ethnicity (Romani/other), sex, weight (in kilograms; time-varying), district of residence (Svidnik/Michalovce), and duration of exclusive breast-feeding (months). **e**Adjusted (as in primary model) results for the subset of infants with thymus measurements performed within 1 week of birth or within 2 weeks of reaching 6 and 16 months of age.				

When we limited our statistical models to the subset of infants with thymus measurements within the first week of birth and within 2 weeks of their 6- and 16-month birthdays, results were generally further away from the null hypothesis (Table 3), although the reduction in sample size also decreased the precision of our estimates. Additionally, infant weight, Romani ethnicity, and male sex were positively associated with thymus volume in our models (data not shown). Duration of exclusive breast-feeding was also positively associated with thymus volume, but only at 6 months of age (data not shown).

When we did not adjust for duration of exclusive breast-feeding, the association between 6-month infant PCB and 6-month thymus was attenuated (0.26-SD reduction for an increase in 6-month infant PCB concentration from the 10th to the 90th percentile; 95% CI: –0.57, 0.06). In addition, when we included breast-feeding duration, time of assessment, and PCB concentration in a three-way interaction model, we did not observe evidence of heterogeneity in the association between PCBs and thymus volume according to breast -feeding status (*p* for interaction = 0.91).

## Discussion

The present study was designed to address two related research questions: first, whether higher *in utero* PCB concentrations are associated with smaller thymus volumes later in infancy; and second, whether infant PCB concentrations measured at 6 and 16 months of age were inversely associated with concurrent thymus volume measurements. Results from our study show that *a*) although higher maternal PCB concentrations are associated with a smaller thymus volume at birth, they are not associated with thymus volume beyond the neonatal period, and *b*) thymus volume at 6 months of age is more strongly associated with concurrently measured infant PCB concentrations than with maternal PCB concentrations.

In this eastern Slovak population, we observed smaller thymus volumes in relation to measures of maternal and infant PCB concentrations, which included predominantly non-dioxin-like congeners. However, much of the experimental literature concerning thymic atrophy and persistent organic pollutants deals with exposure to TCDD and other coplanar PCBs (e.g., PCB-126) as the exposure of interest. In these studies, TCDD acts as a model compound for immune suppression because it strongly binds the aryl hydrocarbon (Ah) receptor (Safe 1994; Silkworth et al. 1984; Tryphonas and Feeley 2001), an intracellular protein that regulates the induction of cytochrome P450 enzymes. The Ah receptor is found in many cells of the immune system, including the thymus, and is fundamental to TCDD-induced immunotoxicity. For instance, mice lacking the Ah receptor have been observed to be highly resistant to the effects of TCDD, exhibiting normal cellular and humoral immune responses (Vorderstrasse et al. 2001) and lacking evidence of thymic atrophy (Fernandez-Salguero et al. 1996). Concentrations of noncoplanar PCBs (which are typically the most prevalent congeners in human serum) are moderately correlated with dioxin-like activity in several populations, including those in eastern Slovakia. For instance, in a study of reproductive-age women in Belgium (Pauwels et al. 2000), serum concentrations of PCB congeners 118, 138, 153, and 180 were each positively correlated, with chemically activated luciferase expression (CALUX) toxic equivalent (TEQ) values of 0.34 ≤ *r* ≥ 0.39, as was the sum of these four congeners (Pearson *r* = 0.43). In serum specimens collected from 300 men and women in eastern Slovakia as part of the PCBRISK project (Petrik et al. 2006), concentrations of PCB congeners 138, 153, 170, and 180 were positively associated with the total WHO (Van den Berg et al. 2006) TEQ (Spearman *r* ≥ 0.58) (Kocan A, personal communication). Thus, although we did not measure coplanar PCB congeners in our study and our analyses focused on the association between thymus volume and noncoplanar PCBs, the moderately strong correlations between non-dioxin-like PCB concentrations and total TEQ in this population suggest that the observed decreases in thymus volume may be the result of dioxin-like activity, mediated through the Ah receptor. Therefore, results from experimental studies involving TCDD (discussed below) may nevertheless be relevant in guiding causal inference.

The first finding from this study indicates that although higher maternal PCBs are associated with reduced thymus size at birth [a finding previously reported by Park et al. (2008) in this cohort)], maternal PCB concentrations are not associated with later thymus measures at 6 and 16 months of age. This finding appears to coincide with the results of experimental studies where pregnant mice were dosed with TCDD during gestation, and thymic development was followed in their offspring. In one of these studies, a single maternal dose of TCDD (10 μg/kg body weight) was given on gestational day 14, and thymic development was assessed on postnatal days (PNDs) 4, 7, 11, and 18 in the pups (Fine et al. 1989). Results showed differences in thymus weight (as a proportion of body weight) and thymic cellularity between treatment and control groups for PNDs 4–11, but by PND18 both thymic weight and cellularity were similar between control and treatment groups. Another study dosed pregnant mice with TCDD on gestational days 6–14 and examined thymic cellularity in offspring at PNDs 6, 14, and 21 and at 7, 8, and 10 weeks (Holladay et al. 1991). Although differences in thymic cellularity were observed on PND6, by PND14 thymic cellularity was similar for treated and control groups. These findings from experimental studies and the results of the present study suggest that the association between *in utero* exposure to dioxin-like compounds and thymic volume may be limited to the neonatal period. However, the impact on functional immunocompetence over time is unclear.

The second finding from our study indicates that 6-month infant PCB concentrations are inversely associated with thymus volume at age 6 months. Moreover, we observed a larger decrease in thymus volume at 6 months associated with the 6-month PCB measure compared with *in utero* PCB exposure. This suggests that the thymus is sensitive to the potential effects of PCBs beyond the prenatal period, a finding also observed in experimental studies of TCDD (Faith and Luster 1979). In our population, thymus size peaked at approximately 6 months of age and then decreased by 16 months of age, consistent with previous studies documenting the development of the thymus in humans (George and Ritter 1996; Hasselbalch et al. 1997; Steinmann et al. 1985). These results suggest that 6 months of age is not only the time of maximum thymus volume during infancy but may also be the period of greatest sensitivity to PCBs. This finding should be interpreted cautiously, however, because our estimate of 6-month infant PCBs and thymus volume was relatively imprecise. In addition, we noted that the association between 6-month infant PCB concentration and 6-month thymus volume was stronger when adjusting for breast-feeding. Because length of exclusive breast -feeding is positively associated with both infant PCB concentration and thymus volume, unadjusted estimates of the PCB–thymus association at 6 months would tend to underestimate the adverse association between infant PCB concentration and thymus volume.

Finally, we did observe some evidence of a positive association between 16-month PCB concentration and 16-month thymus volume. Because the thymus size peaks at approximately 8 months of age and then shrinks (Hasselbalch et al. 1997), a larger thymus volume at 16 months could occur if the shrinkage occurred later than expected, representing delayed maturation of the thymus. That is, infants with a smaller thymic volume at 6 months may reach their peak later, and the decline by 16 months may be less than would occur in those whose thymus reached a peak at the typical time point. The actual functional significance in terms of lymphocyte differentiation and maturation is unclear, and any possible mechanism should be interpreted cautiously given the lack of precision we observed in our estimate of PCBs and thymus volume at 16 months.

Some limitations of our study deserve discussion. First, because of the low concentrations of coplanar PCBs and other dioxin-like compounds in our study and the high cost to quantitate them, we were unable to measure these compounds in maternal and infant sera. Second, only a portion of 6-month infant serum samples was analyzed for PCB concentrations, and although our stratified random sampling scheme had the benefit of increasing statistical power (relative to a similar size sample selected at random), CIs for the estimate of 6-month PCB concentration and 6-month thymus volume indicated that the estimate was relatively imprecise.

The present study has many strengths, which include a biomarker of PCB exposure as opposed to surrogate measures (e.g., breast-feeding duration, fish consumption), as well as repeated assessments of thymus volume during infancy. Repeated measures of both PCB concentration and thymus volume provided more specificity in terms of the timing of exposure and potential adverse outcomes and greater statistical power. To our knowledge, this is the first study to directly assess thymus volume over time in relation to a suspected human toxicant. Finally, the validity of measurements in this cohort is supported by the concordance with the extant literature. Similar to our results, others have found breast-feeding and measures of growth such as birth weight and birth length to be associated with larger thymic index (Benn et al. 2001; Hasselbalch et al. 1997; Iscan et al. 2000; Yekeler et al. 2004).

Taken together, the present study suggests that the association between *in utero* exposure to PCBs and thymus volume may be limited to the neonatal period, although thymus volume measures later in infancy may be affected by postnatal PCB exposures. Whether reductions in thymus volume are associated with compromised immune function or increased morbidity in this population remains to be seen.
